# Associations between neighborhood disadvantage, cannabis use, and patient-reported outcomes among patients with cancer

**DOI:** 10.1016/j.dadr.2026.100449

**Published:** 2026-05-23

**Authors:** Brittney Greene, Shilpa Ghosh, Craig Colder, Salimah Meghani, Brooke Worster, Rebecca Ashare

**Affiliations:** a Department of Psychology at the State University of New York, University at Buffalo, United States; b University of Pennsylvania School of Nursing, United States; c Sidney Kimmel Cancer Center, Thomas Jefferson University, United States

**Keywords:** Cannabis, Cancer, Neighborhood disadvantage, Health outcomes, Cannabis source, Cannabis route

## Abstract

**Introduction:**

Cannabis use is proliferating among oncological populations. However, research is limited on whether cannabis routes and sources are associated with neighborhood disadvantage and whether it moderates the relationship between neighborhood disadvantage and patient-reported outcomes (PROs). This analysis evaluates the relationship between neighborhood disadvantage and cannabis source and route, and their association with PROs.

**Methods:**

Analyses included 106 patients with cancer from varying levels of neighborhood disadvantage. Logistic regressions tested the association of neighborhood disadvantage with cannabis source (i.e., regulated vs. unregulated) and route (i.e., inhaled vs. consumed/other formulations). Multilevel regressions tested the association of neighborhood disadvantage with PROs (e.g., anxiety, depression, sleep quality, pain, quality of life) and moderation by cannabis variables.

**Results:**

Neighborhood disadvantage did not uniquely predict cannabis source or route (*p*’s > .05) or PROs (*p*’s > .05). Conversely, household income predicted pain interference and severity, quality of life, and depression, indicating that lower household income was associated with worse outcomes (*p*’s < .05). There was a significant interaction between neighborhood disadvantage and visit for both pain interference (*b*=-1.37, 95%CI [-2.4,-0.3], *p* = .01) and sleep quality (*b*=1.74, 95%CI [-0.001,3.5], *p* = .05). Cannabis route significantly moderated the relationship between neighborhood disadvantage and anxiety (*b*=-5.32, 95%CI [-10.2,-0.4], *p* = .03). The interaction was driven by reversed scores between patients using inhaled and consumed/other formulations at different levels of neighborhood disadvantage.

**Conclusion:**

Although the composite index of neighborhood disadvantage was not associated with cannabis use or PROs, our data suggest that income and cannabis route may be key factors associated with health disparities among patients with cancer.

## Highlights

• A third of patients with cancer use cannabis as a symptom management option.
• Pain interference and sleep quality scores vary across ADI groups over time.
• Cannabis route affects anxiety differently across ADI groups.
• Higher income was associated with better patient reported outcomes.
• Aspects of neighborhood disadvantage may elucidate cannabis use and health differences.

## 1 Introduction

Medical advancements in cancer treatments have reduced cancer’s health risks; however, access to effective symptom management remains sparse for patients living in disadvantaged areas. Neighborhood disadvantage refers to residential areas that face systemic and structural inequities, including limited access to socioeconomic resources (e.g., adequate healthcare, healthy food) and high concentrations of environmental stressors (e.g., poverty, unemployment) stemming from historic discriminatory practices (Riley, 2018). In the oncology context, neighborhood disadvantage has been negatively associated with adverse health outcomes and suboptimal cancer care (Chiang et al., 2023, Hassan et al., 2023), characterized by lower quality of life and greater time to care (Li et al., 2019, Unger et al., 2021). Accordingly, neighborhood disadvantage may have important implications for patients residing in disadvantaged areas, making it crucial to further delineate its association with patient-reported outcomes (PROs).

Patients living in disadvantaged neighborhoods may be exposed to profound challenges, including lower income and safety concerns. Collectively, these contextual factors are associated with chronic stress (Santiago et al., 2011), compound cancer-related stressors (e.g., screenings) (Kurani et al., 2020), and may in turn exacerbate common cancer-related PROs such as pain (Choi et al., 2023), anxiety and depression (Balogun et al., 2024, Goel et al., 2023, Segrin et al., 2025), and sleep disturbance (Segrin et al., 2025). For instance, mood disturbance may arise due to financial insecurity and having to manage daily stressors (Guan et al., 2022, Ross, 2000). Moreover, environmental factors in disadvantaged neighborhoods, such as crime and noise, may disrupt sleep (Fuller-Rowell et al., 2016, Hill et al., 2009). Additionally, stress associated with living in disadvantaged neighborhoods may contribute to adverse physiological outcomes like increased inflammation, thereby increasing the risk of chronic pain (Hannibal and Bishop, 2014). Furthermore, the influence of socioeconomic factors on PROs may be sustained by limited access to health resources, including inequitable access to pain prescriptions and mental health services for low-income patients (Meghani et al., 2020, Wen et al., 2025). Given the significant impact of neighborhood disadvantage on PROs, it is also essential to investigate its role in contemporary symptom management options, such as cannabis.

One-third of patients use cannabis to manage cancer and cancer-treatment-related symptoms (Ellison et al., 2024). Cannabis has purported medicinal properties; however, findings on its effects have been mixed. Evidence suggests cannabis reduces pain and sleep issues (Blake et al., 2018, Wang et al., 2021), but may have negligible (Crichton et al., 2024, Zaki et al., 2017) or adverse effects on anxiety and mood (Steigerwald et al., 2018, Worster et al., 2022). Indeed, one study (*N* = 267) found that compared to those who did not use cannabis, patients who reported using cannabis to treat physiological and psychological symptoms exhibited greater symptom severity on these outcomes (Azizoddin et al., 2023), which is in contrast to reported symptom improvement among patients using cannabis in other studies (McClure et al., 2023, Salz et al., 2023). In light of these mixed findings, the emerging literature has increasingly focused on examining sociodemographic and neighborhood-level factors that may help elucidate the complex relationship between cannabis use and PROs.

Prior work indicates that underserved communities may be more likely to engage in cannabis use patterns associated with higher risk, which may be driven by neighborhood-level factors (Ashare et al., 2023, Azizoddin et al., 2023, Jeffers et al., 2021). For instance, Black patients may be more likely to purchase cannabis from non-licensed retailers (Ashare et al., 2023), and these retailers are more prevalent in disadvantaged neighborhoods (Rhew et al., 2022). Moreover, low-income patients are affected by cannabis pricing and accessibility (Ashare et al., 2023). This has important implications, as obtaining cannabis from non-licensed retailers, likely because of accessibility, may confer a greater risk of exposure to harm due to the lack of regulatory oversight. Additionally, combustible cannabis formulations, which are more prevalent in socioeconomically disadvantaged neighborhoods (Brasky et al., 2025), may increase the risk of carcinogen exposure (Jett et al., 2018). Taken together, neighborhood disadvantage may be associated with cannabis source and route, and these cannabis-related variables may disproportionately affect patients living in disadvantaged neighborhoods due to a theorized elevated risk of cannabis-related adverse outcomes stemming from limited product safety and established health risks.

These adverse outcomes may also manifest in PROs; however, this topic remains understudied in oncology populations. Further research would be beneficial for clarifying the role of cannabis in PROs and for further delineating how the influence of neighborhood disadvantage on PROs differentially affects patients residing in disadvantaged neighborhoods compared with those who do not. To address this gap, the present study evaluates whether neighborhood disadvantage is associated with cannabis source or route and whether these cannabis variables modify the association between neighborhood disadvantage and PROs. We utilized the ADI (2023) to investigate the association of neighborhood disadvantage with cannabis route (i.e., inhaled [smoked, vaped], consumed/other [e.g., topicals, pills]) and source (i.e., regulated [licensed dispensaries] vs. unregulated [family and friends, unlicensed sellers, Native American reservations, smoke shops]). Separately, we assessed the relationship between ADI and key PROs (i.e., pain, sleep quality, anxiety, depression, and quality of life) across time (i.e., visit; coded Month 0, 1, 2, and 3). We hypothesized that greater neighborhood disadvantage would be associated with: (1) obtaining cannabis from unregulated versus regulated sources; (2) using inhaled formulations compared to consumed/other formulations; and (3) greater symptom severity in PROs and greater increases in symptom severity in PROs across time. An exploratory aim tested whether cannabis source and route moderated the association between neighborhood disadvantage and PROs. Additionally, we assessed the relationship between individual socioeconomic characteristics (that were retained as covariates) and PROs.

## 2 Methods

### 2.1 Study design, population, & setting

This is a secondary analysis of an ongoing multi-site study (University at Buffalo [UB], University of Pennsylvania [UP], Thomas Jefferson University [TJU], NCT06037681) investigating the association among PROs and opioid and cannabis use over 12 months. Electronic medical records (EMR) were used to identify potentially eligible participants. Additional recruitment methods included flyers and the Buffalo Research Registry. Data collection began in March 2023. Participants in the current sample were scheduled to complete Month 3 of the parent study by March 28, 2025 and were recruited from the overall study sample at this time (*N* = 361). This study was approved by the UB Institutional Review Board.

Eligible participants were at least 21 years old (the legal age to purchase cannabis), receiving outpatient treatment for solid tumors, and self-reported cancer or cancer treatment-related pain for greater than 30 days in the past three months. Skin cancer was the only cancer diagnosis excluded. At baseline (Month 0), patients were categorized into one of two groups: people who used cannabis (self-reported at least weekly cannabis use over the past month) and people who did not use cannabis (self-reported no cannabis use in the past three months). The parent study aimed to recruit a sample representing 50% of each group.

The present analyses were restricted to the first four months of data collection (Month 0 – Month 3). Participants were included if they used cannabis at baseline, had at least one time point with cannabis route and source information to assess between-subject differences, and at least two time points for one of the PRO variables to identify change over time.

### 2.2 Study procedures

Participants completed an initial screening to determine eligibility. Eligible participants completed a baseline visit, including informed consent and coordinator-administered surveys. Participants then completed surveys at monthly study visits and seven days of ecological momentary assessment (EMA) at each time point (4 surveys/per day: 1 morning and 3 random surveys) to assess PROs, cannabis, and opioid use. Following baseline, EMA data were collected for one week per month for 3 months (total of 21 days). EMAs were administered either through a smartphone-based application (mEMA; ilumivu, Inc) or via REDCap links sent through a third-party application (e.g., Mosio, Inc).

### 2.3 Measures

#### 2.3.1 Sociodemographic and clinical data

Sociodemographic variables (e.g., race, income) were ascertained at baseline. Medical data (e.g., cancer stage, treatment status, cancer type) were obtained from EMRs.

#### 2.3.2 Cannabis use

Cannabis Frequency. Past month average cannabis frequency was collected during monthly study visits.

Cannabis Source and Route. Categorization of cannabis route (i.e., inhaled vs. consumed/other route) was based on both prior literature (Ellison et al., 2024) and observed patterns in the current data. Categorization of cannabis source (i.e., regulated vs unregulated) was based on two primary reasons: (1) dispensaries are the most common source of obtaining cannabis (42.5%) (Myers et al., 2024); (2) and are the only regulated cannabis source.

Data on cannabis source and route were collected at monthly study visits and EMAs. Source and route were first determined via EMA data to reduce potential recall bias. If EMA data were missing, visit data were used. For participants who reported obtaining cannabis from more than one source, the source that was reported most frequently was designated as the primary cannabis source. In cases where there was an equal number of regulated and unregulated sources, the regulated source was selected as the primary source based on the premise that access to regulated sources may reflect higher levels of accessibility. A similar process was conducted to identify the primary route. If routes were reported with equal frequency, inhaled formulations took precedence over other methods based on literature indicating that inhaled formulations may be associated with worse health outcomes (Jett et al., 2018). Few participants showed within-person variability in cannabis source (16.0%) and route (29.3%), reflecting that the majority of participants didn’t change their route or source over time.

Cannabis Use Disorder Identification Test- Revised (CUDIT-R). The 8-item CUDIT-R assessed risk for hazardous cannabis use (Adamson et al., 2010, Adamson and Sellman, 2003). Scores range from 0 to 32, with a score of 8 or more indicating “hazardous cannabis use” and 13 suggesting “possible cannabis use disorder.” Internal consistency in our study was poor (Cronbach’s α=0.55) but excellent in prior studies (Cronbach’s α=0.91) (Adamson et al., 2010).

#### 2.3.3 Neighborhood disadvantage

The Area Deprivation Index (ADI) is a well-validated measure of neighborhood disadvantage and uses 17 metrics from census-tract data to produce a composite score (Kind and Buckingham, 2018, Kind et al., 2014). In prior oncology studies, the ADI has been linked to PROs such as anxiety and has informed recommendations for its use in oncology populations, including recommended screening for neighborhood-level socioeconomic issues and assisting with patient navigation to improve targeted patient care (Rosenzweig et al., 2021). The ADI defines a neighborhood as a census tract, which was identified here by entering an individual’s full address (including zip code) into the ADI’s website. Neighborhood disadvantage is quantified by the ADI’s national decile, which is a composite score that combines key socioeconomic indicators (e.g., income, education) and is weighted and standardized to produce an overall score for each neighborhood (0–100 %ile; higher scores, greater disadvantage). As in prior work (Goel et al., 2023), we used a categorical ADI in all analyses, which partitioned participants’ national deciles into three tertiles (0–33.3% Least Disadvantaged; 33.3–66.6% Moderately Disadvantaged; 66.6–100% Most Disadvantaged).

#### 2.3.4 Patient-reported outcomes

All PROs data were collected from study visits.

Pain. The Brief Pain Inventory Short Form (BPI-SF) assesses clinical pain (Caraceni et al., 1996, Cleeland, 1991). It includes two domains: pain severity and interference with sub-items scored on a scale from 0 (no pain/no interference) to 10 (worse pain imaginable/complete interference). In our study, internal consistency was excellent (Cronbach α=.95).

Sleep Quality. The Pittsburgh Sleep Quality Index (PSQI) is a 9-item measure that assesses sleep quality and patterns over the past month. The outcome is the global PSQI score, where a sum of 5 or greater indicates poor sleep (Buysse et al., 1989). The PSQI had poor internal consistency in our study (Cronbach α=.63), likely due to the variable sleep quality and duration patients with cancer often experience.

Anxiety. The Generalized Anxiety Disorder-7 (GAD-7) is a 7-item measure that assesses clinical severity of anxious symptoms (Spitzer et al., 2006). Total scores ranged from 0 to 21, with the clinical threshold being a score of 10 or higher. Internal consistency in our study was excellent (Cronbach α=.93).

Depression. The Patient Health Questionnaire-8 (PHQ-8) is an 8-item measure of clinical depression (Kroenke et al., 2009). The scaling is the same as the GAD-7. Total scores ranged from 0 to 24, with a clinical threshold of 10 or higher. Internal consistency in our study was good (Cronbach α=.88).

Quality of Life. The Functional Assessment of Cancer Therapy-General 7 (FACT-G7) scale is a 7-item cancer-specific quality of life index adapted from the 27-item FACT-G (Cella et al., 1993, Mah and Swami, 2020). In our study, internal consistency was excellent (Cronbach α=.91). It has also been shown to decrease with worsening disease in patients with cancer (Pearman et al., 2014).

#### 2.3.5 Other substance use

Opioid Use. Opioids are frequently prescribed to patients with cancer for pain management (Mudumbai et al., 2024). We assessed opioid utilization by querying participants about their opioid consumption during the past month before each visit. Responses were coded as 1 = ‘yes’ or 0 = ‘no’.

Alcohol and Tobacco Use. Because alcohol and tobacco use correlate with cannabis use (Lemyre et al., 2019, Yurasek et al., 2017), these were measured at baseline as statistical control variables. The Alcohol Use CAGE is a 4-item, self-report measure of alcohol dependence (Ewing, 1984) with high-test retest reliability (.80–.95) and adequate correlations (.48–.70) with other screening instruments (Dhalla and Kopec, 2007). Participants reported past-month tobacco use during visits. No patients reported current alcohol or tobacco use; consequently, these variables were not included in analyses.

### 2.4 Data analysis

#### 2.4.1 Statistical power

Because the goal of this study is to generate data to inform future studies, a power analysis was not conducted.

#### 2.4.2 Statistical analysis

Descriptive statistics were calculated to compare patients across ADI tertiles using bivariate analyses (Chi-square for categorical data; one-way ANOVAs for continuous data). Analyses that violated Bartlett’s assumption of equal variances were then analyzed using non-parametric tests. Covariates associated with study outcomes with a *p* < .1 were retained in subsequent models. For all analyses, including neighborhood disadvantage, the reference group was the most disadvantaged group. Logistic regressions were used to determine whether neighborhood disadvantage was associated with primary cannabis source (regulated vs. unregulated) and route (inhaled vs. consumed/other), respectively. Because few patients showed within-person variability for cannabis variables, the logistic regression analyses modeled between-person variability only.

The PROs data conformed to a multilevel structure with observations at each visit nested within patients. We estimated two-level mixed-effect models with neighborhood disadvantage as a categorical predictor to describe its association with each PRO, including random effects of visit. A series of models were fitted to identify the most appropriate model for each PRO. First, we estimated the intercept-only model for each PRO and then added patient as a random effect to evaluate differences in baseline-level outcomes. Next, we added visit (i.e., time, coded Month 0, 1, 2, 3) as a fixed effect to evaluate the average change in a specified PRO and then added a random effect for visit to examine individual differences in change. Likelihood-ratio tests were used to determine the best-fitting model for each PRO. Results yielded a random-intercept only model for anxiety and depression, a random intercept and fixed effect of visit for pain interference, sleep quality, and quality of life, and a random intercept and random effect of visit for pain severity. All models used maximum likelihood estimation. Models with pain severity as the outcome had an unstructured covariance structure to allow for correlations between random effects. Final models included all study covariates (race, income, and site).

We tested the interaction between neighborhood disadvantage and visit predicting PROs when nested model tests supported the inclusion of visit. Exploratory analyses included testing the interaction between neighborhood disadvantage and cannabis source and route, respectively, on each PRO. Post hoc analyses for covariates were also conducted.

## 3 Results

### 3.1 Sociodemographic, cannabis, and clinical characteristics

**Fig. 1:**
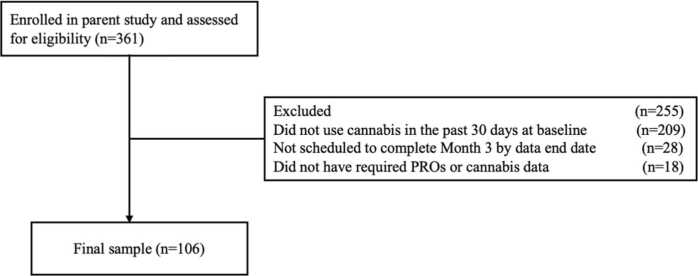
Participant Flowchart.

Table 1 contains sociodemographic and cannabis use characteristics by neighborhood disadvantage. Fig. 1 outlines the participant flowchart. The sample comprised 106 patients with cancer and was majority female (56.6%), white (65.1%), retired or disabled (58.5%), low (<$35,000) to lower-middle-income ($35,000-$99,000; 79.1%), and used cannabis daily or more (61.9%). Relative to those in the least and moderately disadvantaged groups, more patients from the most disadvantaged group were African-American/Black (*p* < .001), low-income (*p* < .01), and found it difficult to get by on their current income (*p* = .01). UB enrolled more patients from the most disadvantaged group than other sites (*p* < .01). Additional characteristics were similar across groups (see Table 1 and Supplemental Table 1).

**Table 1:** Sociodemographic and cannabis characteristics by neighborhood disadvantage.

Characteristics			Most Disadvantaged	Moderately Disadvantaged	Least Disadvantaged	Overall	*p*-value
			(n = 44)	(n = 34)	(n = 28)	(n = 106)	
Site n (%)							**< .01**
	University at Buffalo (UB)		18 (40.9%)	16 (47.1%)	3 (10.7%)	37 (34.9%)	
	University of Pennsylvania (UP)		10 (22.7%)	6 (17.7%)	16 (57.1%)	32 (30.2%)	
	Thomas Jefferson University (TJU)		16 (36.4%)	12 (35.3%)	9 (32.1%)	37 (34.9%)	
Age mean (SD)			54 (13)	55 (12)	55 (11)	55 (12)	.88
Sex n (female %)			24 (54.6%)	22 (64.7%)	14 (50.0%)	60 (56.6%)	.48
Race n (black %)			27 (61.4%)	6 (17.7%)	4 (14.3%)	37 (34.9%)	**< .001**
State n (%)							**.04**
	Pennsylvania		23 (52.3%)	13 (38.2%)	22 (78.6%)	58 (54.7%)	
	New York		18 (40.9%)	16 (47.1%)	3 (10.7%)	37 (34.9%)	
	New Jersey		2 (4.6%)	4 (11.8%)	3 (10.7%)	9 (8.5%)	
	Other		1 (2.3%)	1 (2.9%)	0 (0.0%)	2 (1.9%)	
Marital Status n (%)							.24
	Married or Cohabitating		17 (38.6%)	20 (58.8%)	17 (60.7%)	54 (50.9%)	
	Divorced, Separated, or Widowed		13 (29.6%)	9 (26.5%)	6 (21.4%)	28 (26.4%)	
	Never Married		14 (31.8%)	5 (14.7%)	5 (17.9%)	24 (22.6%)	
Education n (%)							.17
	High School Degree or Lower		13 (29.6%)	4 (12.1%)	4 (14.3%)	21 (20.0%)	
	Some College/Technical School		20 (45.5%)	15 (45.5%)	11 (39.3%)	46 (43.8%)	
	College Graduate or Greater		11 (25.0%)	14 (42.4%)	13 (46.4%)	38 (36.2%)	
Employment n (%)							.23
	Employed		8 (18.2%)	9 (26.5%)	9 (32.1%)	26 (24.5%)	
	Retired or Disabled		32 (72.7%)	17 (50.0%)	13 (46.4%)	62 (58.5%)	
	Unemployed		4 (9.1%)	6 (17.7%)	4 (14.3%)	14 (13.2%)	
	Other		0 (0.0%)	2 (5.9%)	2 (7.1%)	4 (3.8%)	
Health Insurance n (%)							.22
	Employer Plan		10 (22.7%)	14 (41.2%)	13 (46.4%)	37 (34.9%)	
	Private		2 (4.6%)	2 (5.9%)	3 (10.7%)	7 (6.6%)	
	Medicaid/Medicare		29 (65.9%)	16 (47.1%)	12 (42.9%)	57 (53.8%)	
	Other		3 (6.8%)	2 (5.9%)	0 (0.0%)	5 (4.7%)	
Income n (%)							**.01**
	Less than $35,000		25 (56.8%)	10 (30.3%)	5 (17.9%)	40 (38.1%)	
	$35,000 - $99,000		15 (34.1%)	15 (45.5%)	13 (46.4%)	43 (41.0%)	
	$100,000 or Greater		4 (9.1%)	8 (24.2%)	10 (35.7%)	22 (21.0%)	
Income Satisfaction n (%)							**.01**
	Living Comfortably on Current Income		4 (9.1%)	12 (36.4%)	13 (46.4%)	29 (27.6%)	
	Getting by on Current Income		22 (50.0%)	8 (24.2%)	9 (32.1%)	39 (37.1%)	
	Finding it Difficult on Current Income		13 (29.6%)	7 (21.2%)	4 (14.3%)	24 (22.9%)	
	Finding it Very Difficult on Current Income		5 (11.4%)	6 (18.2%)	2 (7.1%)	13 (12.4%)	
Cannabis Use Frequency n (%)							.96
	A Few Times a Month		3 (6.8%)	2 (6.1%)	3 (10.7%)	8 (7.6%)	
	Once to Several Days a Week		13 (29.6%)	10 (30.3%)	9 (32.1%)	32 (30.5%)	
	Daily or More		28 (63.6%)	21 (63.6%)	16 (57.1%)	65 (61.9%)	
Used Multiple Cannabis Routes n (yes %)			16 (36.4%)	9 (26.5%)	6 (21.4%)	31 (29.3%)	.36
Went to Multiple Cannabis Sources n (yes %)			8 (18.2%)	5 (14.7%)	4 (14.3%)	17 (16.0%)	.88
Cannabis Dependence mean (SD)			7 (4.0)	7 (3.0)	5 (2.0)	6 (3.0)	.10
Opioid Prescription Receipt n (yes %)			27 (61.4%)	23 (67.7%)	23 (82.1%)	73 (68.9%)	.18

**Note.** Bolded results represent associations significant at the *p* < .05 level. No reported income or education data for one participant. Categorical variables were tested with chi-square and continuous variables were tested with ANOVA. All p-values reflect the omnibus test.

### 3.2 Cannabis source and route

#### 3.2.1 Cannabis source

**Fig. 2:**
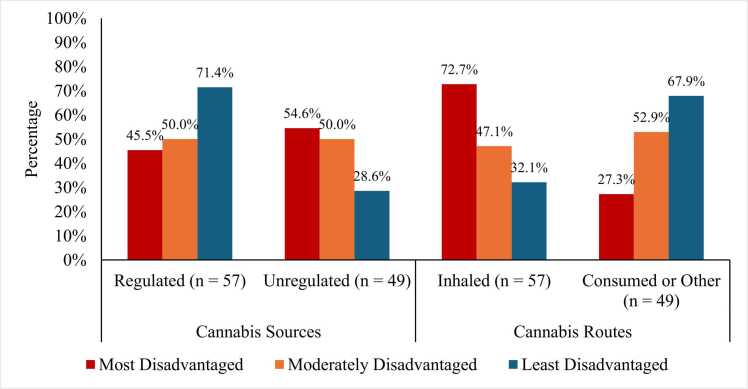
Cannabis sources and routes by neighborhood disadvantage.

A majority of participants reported obtaining cannabis from regulated versus unregulated sources (53.8% vs. 46.2%). Cannabis source by ADI groups is reflected in Fig. 2. The odds of obtaining cannabis from regulated versus unregulated sources were comparable between patients from the least and most disadvantaged groups (OR=0.92, 95%CI [0.2,3.5], *p* = .90) and patients from moderately and most disadvantaged groups (OR=0.84, 95%CI [0.3,2.6], *p* = .75). Patients enrolled at UP (OR=8.27, 95%CI [2.2,30.5], *p* < .01) or TJU (OR=6.46, 95%CI [1.9,21.4], *p* < .01) were significantly more likely to obtain cannabis from regulated sources compared to patients from UB. Patients who were middle-upper-income (>$100,000) and lower-middle-income were more likely to obtain cannabis from regulated sources compared to low-income patients (OR=5.3, 95%CI [1.3,22.1], *p* = .02; OR=2.9, 95%CI [1.0,8.3], *p* = .05, respectively).

#### 3.2.2 Cannabis route

Most participants reported using inhaled cannabis formulations compared to consumed/other formulations (53.8% vs. 46.2%). Shown in Fig. 2, patients from the least and most disadvantaged groups (OR=2.51, 95%CI [0.7,8.7], *p* = .15) and moderately and most disadvantaged groups (OR=2.10, 95%CI [0.7,6.2], *p* = .18) had comparable likelihood of using consumed/other versus inhaled formulations. Compared to low-income patients, lower-middle-income patients were 4.1 times more likely to use consumed/other formulations than inhaled (95%CI [1.4,11.4], *p* = .01); however, this effect was not observed for middle-higher-income patients (OR=2.89, 95%CI [0.8,10.5], *p* = .11).

### 3.3 Multilevel regressions predicting PROs

Main Effects. The main effect of neighborhood disadvantage was non-significant in all models, indicating that patients in the moderately or least disadvantaged groups did not differ in mean PRO scores from patients in the most disadvantaged group (*p*’s = .13–.79 and.27–.96, respectively). Visit was significant for pain interference (*b*=-0.18, 95%CI [-0.3,-0.04], *p* = .01), sleep quality (*b*=-0.34, 95%CI [-0.6,-0.1], *p* < .01), and marginal for quality of life (*b*=0.69, 95%CI [-0.05,1.4], *p* = .07). On average, outcomes improved over time (i.e., lower pain interference and higher sleep quality and quality of life). Patient income was a significant predictor in all models, and marginal for sleep quality and anxiety. Compared to the low-income group, being lower-middle-income or middle-upper-income was associated with better outcomes for pain interference and severity, quality of life, and depression (*p*’s = .01–.03 and <.001–.04, respectively). Associations for neighborhood disadvantage and all covariates with PROs are depicted in Table 2.

**Table 2:** Main effects of neighborhood disadvantage and covariates on patient-reported outcomes.

			β	95% CI	*p*-value
Pain Interference					
	Neighborhood Disadvantage				
		Moderately Disadvantaged	-0.81	(-1.8,0.2)	.13
		Least Disadvantaged	-0.29	(-1.5,0.9)	.64
	Income				
		$35,000 - $99,000	-1.14	(-2.1,-0.2)	**.02**
		$100,000 or Greater	-2.35	(-3.6,-1.1)	**< .001**
	Race				
		African American/Black	-0.2	(-1.3,0.9)	.71
	Site				
		University of Pennsylvania (UPenn)	0.77	(-0.4,1.9)	.19
		Thomas Jefferson University (TJU)	0.69	(-0.4,1.7)	.20
Pain Severity					
	Neighborhood Disadvantage				
		Moderately Disadvantaged	-0.23	(-1.6,0.7)	.63
		Least Disadvantaged	0.09	(-1.0,1.2)	.86
	Income				
		$35,000 - $99,000	-1.11	(-2.0,-0.3)	**.01**
		$100,000 or Greater	-2.19	(-3.3,-1.1)	**< .001**
	Race				
		African American/Black	0.91	(-0.05,1.9)	.06
	Site				
		University of Pennsylvania (UPenn)	0.80	(-0.2,1.8)	.13
		Thomas Jefferson University (TJU)	0.38	(-0.6,1.3)	.43
Sleep Quality					
	Neighborhood Disadvantage				
		Moderately Disadvantaged	0.59	(-1.1,2.3)	.49
		Least Disadvantaged	-0.05	(-2.0,1.9)	.96
	Income				
		$35,000 - $99,000	-1.49	(-3.0,0.05)	.06
		$100,000 or Greater	-1.72	(-3.7,0.3)	.10
	Race				
		African American/Black	0.24	(-1.5,2.0)	.79
	Site				
		University of Pennsylvania (UPenn)	-0.04	(-1.9,1.8)	.97
		Thomas Jefferson University (TJU)	0.07	(-1.6,1.8)	.94
Quality of Life					
	Neighborhood Disadvantage				
		Moderately Disadvantaged	4.96	(-3.6,13.5)	.26
		Least Disadvantaged	5.51	(-4.2,15.3)	.27
	Income				
		$35,000 - $99,000	8.46	(0.7,16.3)	**.03**
		$100,000 or Greater	10.67	(0.5,20.8)	**.04**
	Race				
		African American/Black	3.1	(-5.8,12.0)	.49
	Site				
		University of Pennsylvania (UPenn)	-0.62	(-10.1,8.8)	.90
		Thomas Jefferson University (TJU)	-4.77	(-13.3,3.7)	.27
Anxiety					
	Neighborhood Disadvantage				
		Moderately Disadvantaged	-0.34	(-2.9,2.2)	.79
		Least Disadvantaged	-1.24	(-4.1,1.7)	.41
	Income				
		$35,000 - $99,000	-2.09	(-4.5,0.3)	.09
		$100,000 or Greater	-1.20	(-4.3,1.9)	.44
	Race				
		African American/Black	-1.4	(-4.1,1.2)	.28
	Site				
		University of Pennsylvania (UPenn)	-0.50	(-3.3,2.3)	.73
		Thomas Jefferson University (TJU)	2.41	(-0.1,4.9)	.06
Depression					
	Neighborhood Disadvantage				
		Moderately Disadvantaged	-0.94	(-3.6,1.8)	.50
		Least Disadvantaged	-0.91	(-4.1,2.3)	.58
	Income				
		$35,000 - $99,000	-3.16	(-5.6,-0.7)	**.01**
		$100,000 or Greater	-3.54	(-6.7,-0.3)	**.03**
	Race				
		African American/Black	-1.12	(-3.9,1.7)	.43
	Site				
		University of Pennsylvania (UPenn)	-0.29	(-3.3,2.8)	.85
		Thomas Jefferson University (TJU)	1.91	(-0.7,4.6)	.16

**Note.** Bolded results represent associations significant at the *p* < .05 level. References groups are as follows: Neighborhood Disadvantage - Most Disadvantaged, Income - $35,000 or less, Race - White, Site - University of Buffalo (UB).

**Fig. 3:**
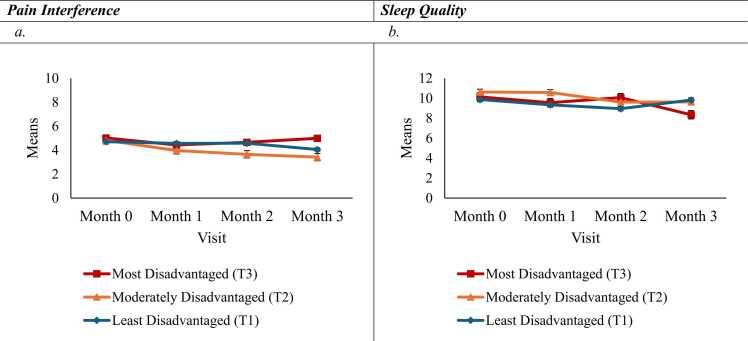
Interaction Effects of Neighborhood Disadvantage and Visit on Pain Interference and Sleep Quality.

Interactions. There was a significant interaction between neighborhood disadvantage and visit for both pain interference (*b*=-1.37, 95%CI [-2.4,-0.3], *p* = .01) and sleep quality (*b*=1.74, 95%CI [-0.001,3.5], *p* = .05). For pain interference, patients in the moderately disadvantaged group exhibited greater declines in pain interference scores over time, with significantly lower levels at Month 3 compared to those in the most disadvantaged group (Fig. 3a). In contrast, the most disadvantaged group demonstrated minimal change across visits. Simple effects analyses indicated that patients in the moderately disadvantaged group experienced reductions in pain interference scores at all time points (*p = .*001–.03). For sleep quality, there was overall improvement over time among patients in the most disadvantaged group; however, this improvement was attenuated among those in the least disadvantaged group (Fig. 3b). Indicated by simple effects, there were improvements in sleep disturbance at Month 3 for patients in the most disadvantaged group (*p* < .05), with smaller changes observed in the least disadvantaged group. The interaction was non-significant for pain severity (*p* = .26–.97) and quality of life (*p* = .13–.69). Because the best-fitting model for anxiety, depression, and pain severity did not include visit as a predictor, the interaction was not tested.

### 3.4 Cannabis source added to main effect and interaction models

Main Effects. Cannabis source was not a significant predictor for any PRO (*p*’s = .24–.93). Adding cannabis source did not change the main effects of visit and income and marginal effects of race described above and in Table 2. However, when controlling for cannabis source, race significantly predicted pain severity, where Black patients had significantly higher pain severity scores compared to White patients (*b*=0.94, 95%CI [-0.01,1.9], *p* = .05). Additionally, site significantly predicted anxiety such that patients from TJU had significantly higher anxiety scores than patients recruited from UB (*b*=2.63, 95%CI [-0.03,5.3], *p* = .05).

Interactions. The neighborhood disadvantage x cannabis source interaction was non-significant across all models (*p*’s = .14–.81).

### 3.5 Cannabis route added to main effect and interaction models

Main Effects. Cannabis route was not a significant predictor for any PRO (*p*’s = .40–.76) and adding it to the model did not significantly change prior main effects of visit and covariates.

**Fig. 4:**
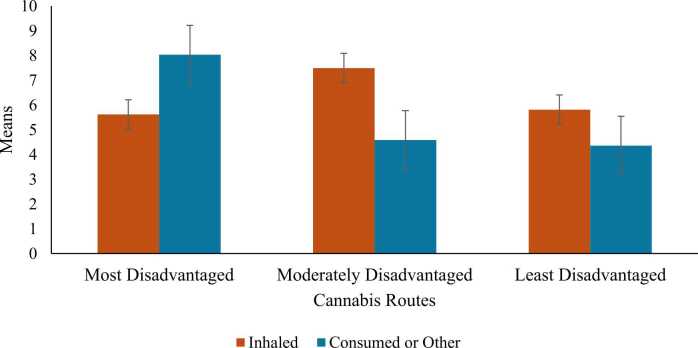
Interaction Effects of Neighborhood Disadvantage and Cannabis Route on Anxiety.

Interactions. There was a significant neighborhood disadvantage x cannabis route interaction on anxiety scores, across levels of neighborhood disadvantage (*b*=-5.32, 95%CI [-10.2,-0.4], *p* = .03). For patients using inhaled formulations, moderate levels of neighborhood disadvantage were associated with higher anxiety compared to the highest level of neighborhood disadvantage (Fig. 4). In contrast, among patients using consumed/other formulations, this association was reversed, as moderate levels of neighborhood disadvantage were associated with lower anxiety scores. Simple effects were non-significant (*p* = .12–.51). The interaction effect was non-significant in all other models (*p*’s = .17–.97).

### 3.6 Supplemental analyses

Income is a significant patient-level factor that unveils health disparities, is an indicator of neighborhood disadvantage, and was associated with study outcomes. Therefore, we assessed its relationship with sociodemographic, cannabis, and clinical variables in supplemental analyses.

## 4 Discussion

Our study explored differences in cannabis route and source and PROs among patients with cancer across varying levels of neighborhood disadvantage. Changes in pain interference and sleep quality scores over time differed between levels of neighborhood disadvantage. Cannabis route moderated the relationship between neighborhood disadvantage and anxiety. Inconsistent with the extant literature, neighborhood disadvantage was not associated with our outcomes when accounting for individual characteristics. Given the novelty of exploring cannabis use among oncology populations and its relationship with neighborhood disadvantage, these findings may guide future oncology studies on PROs and cannabis access.

We assessed the relationship between neighborhood disadvantage and cannabis route, given that SDoH are associated with cannabis access (Ashare et al., 2023) and that inhaled cannabis formulations may pose potential health risks (Jett et al., 2018). Although non-significant, our findings were in the predicted direction as patients from more advantaged neighborhoods were more likely to use consumed/other formulations than inhaled, compared to those from the most disadvantaged neighborhoods. This aligns with literature suggesting that inhaled formulations are more prevalent in higher deprivation areas (Brasky et al., 2025) and that engaging in risky substance use behaviors is more common among patients facing greater social disparities (Simmons et al., 2016). Additionally, inhaled formulations may cost less, enhancing accessibility to more disadvantaged patients.

Our findings indicate that patients in the moderately disadvantaged group showed greater improvements in pain interference scores over time than those in the most disadvantaged group. This pattern is consistent with prior literature suggesting that residence in impoverished neighborhoods is associated with more pronounced pain disparities (Maly and Vallerand, 2018). Additionally, salient sociodemographic characteristics may have differentiated these groups. Black and low-income patients disproportionately experience more severe pain-related disparities, which are linked to limited access to care (Meghani et al., 2020, Youn et al., 2024). In our sample, patients in the moderately disadvantaged group were predominantly White and had higher incomes than the most disadvantaged group, who were largely Black and low-income. Consequently, when experiencing pain interference, they may have had greater socioeconomic resources to facilitate symptom management changes compared with the most disadvantaged group. The present findings further substantiate this hypothesis. In our sample, higher income was uniquely associated with lower average pain interference scores, and prior research has similarly identified this association (Peoples et al., 2024). In addition to pain interference, there were also group differences in sleep quality scores over time. Patients in the least disadvantaged group demonstrated worse sleep quality scores over time relative to those in the most disadvantaged group, a pattern that is inconsistent with existing literature (Fuller-Rowell et al., 2016, Troxel et al., 2018). Further investigation of clinical, sociodemographic, and cannabis-related factors may help to elucidate these unexpected findings.

Patients using inhaled formulations and residing in moderately disadvantaged neighborhoods had higher anxiety scores than those residing in the most disadvantaged neighborhoods; this relationship was reversed in patients using consumed/other formulations. While existing literature links neighborhood disadvantage to anxiety (Balogun et al., 2024), research on cannabis’ impact on anxiety remains mixed (Van Ameringen et al., 2020). Several factors may explain these findings, such as patient-level factors, including income and cannabis use patterns, like quantities consumed (Ko et al., 2016). Patients who use consumed/other formulations and reside in disadvantaged neighborhoods may be under-consuming these products or taking an ineffective dose, given their higher price point and limited access. Inhaled formulations may be used more consistently among patients residing in the most disadvantaged neighborhoods due to their affordability and accessibility, likely reducing their anxiety. Conversely, patients using consumed/other formulations residing in moderately disadvantaged neighborhoods had lower anxiety scores than those in the most disadvantaged group. Understanding cannabis characteristics like dosage and effectiveness, and their relation to socioeconomic factors, may help delineate these trends in anxiety. These postulations are speculative, and further research is required.

The interaction between neighborhood disadvantage and cannabis route was non-significant for all other PROs. Given that all participants used cannabis, this may have attenuated our ability to detect effects due to self-medication of PROs with cannabis use, but this was not tested, given the absence of a control group who did not use cannabis. Meghani and colleagues (2021) found that racial disparities in self-reported pain among patients with cancer were no longer significant after accounting for cannabis use. Thus, in our study, cannabis may have minimized potential disparities in PROs.

Descriptively, more patients from the least disadvantaged group obtained cannabis from regulated sources than those from moderately and most disadvantaged groups. Controlling for covariates, this effect dissipated. Since neighborhood disadvantage did not independently predict this or other outcomes in our study, it is important to explore how patient-level factors may contribute to cannabis access disparities. For instance, dispensaries are typically more expensive than unlicensed markets because of their higher-priced products and greater cannabis tax on people who purchase cannabis (Han and Shi, 2025). Thus, income may explain our descriptive differences in cannabis source across levels of neighborhood disadvantage. This is supported by our finding that income independently predicted cannabis source and route and PROs, consistent with prior studies (Walker et al., 2021, Youn et al., 2024). Thus, income should be explored as a predictor in future work to better contextualize differences in cannabis use and PROs among patients with cancer. Additionally, given that household income is an indicator of neighborhood disadvantage, exploring income as a community-level factor may help explain differences across levels of neighborhood disadvantage.

### 4.1 Limitations

Several limitations should be considered. Since ADI uses census-tract data, it may provide similar scores to neighborhoods that share a zip code, not accounting for potential variability in SDoH that differentiates them. Our sample size may not have been adequate to identify many possible associations from patient-level ADI indicators, especially in multilevel analyses. Thus, future studies should recruit larger samples. Similarly, there was less representation from the least disadvantaged group in the current sample, which may have skewed analyses. All data were self-reported and subject to recall bias. Similarly, some patients may have been unable to discern between regulated (e.g., licensed dispensaries) and unregulated sources (e.g., smoke shops selling cannabis). Indeed, a previous study found that participants misclassified nicotine smoke and vape shops (Giovenco, 2018). Although descriptive differences between the least and most disadvantaged groups on cannabis source in our study suggest this may not have posed as a substantial limitation, it should be investigated in future work. Lastly, our findings may not be generalizable to oncology populations residing outside of the East Coast, who may have distinct structural differences that contribute to neighborhood disadvantage, or general populations who may differ in symptom severity and cannabis-use patterns from oncology populations.

## 5 Conclusion

Our study contributes to the emerging literature on cannabis use and inequities in oncology populations. Preliminary findings suggest that sociodemographic factors, like income, may help elucidate observed differences in PROs. Cannabis route emerges as a critical variable warranting further investigation, as it may explain variations in PROs and be a target for future intervention research. Future studies should examine behavioral patterns associated with cannabis use to enhance our understanding of the variability in PROs and cannabis consumption among patients with cancer.

## Contributors

BG, RA, and SM developed the manuscript concept. BG and SG conducted data analysis. All authors drafted and edited the initial manuscript. All authors approved the final version of the manuscript for submission.

## CRediT authorship contribution statement

**Shilpa Ghosh:** Writing – review & editing, Methodology, Formal analysis. **Brittney Greene:** Writing – review & editing, Writing – original draft, Visualization, Methodology, Funding acquisition, Formal analysis, Conceptualization. **Rebecca Ashare:** Writing – review & editing, Methodology, Funding acquisition, Conceptualization. **Brooke Worster:** Writing – review & editing, Funding acquisition. **Salimah Meghani:** Writing – review & editing, Funding acquisition. **Craig Colder:** Writing – review & editing, Methodology.

## Declaration of Generative AI and AI-assisted technologies in the writing process

During the preparation of this work the authors used Grammarly (Grammarly.com) to assist with grammar, spell-check, and clarity. After using this tool, the authors reviewed and edited the content as needed and takes full responsibility for the content of the published article.

## Role of funding source

This research was supported by the 10.13039/100000054National Cancer Institute under award number R01CA270483–02S1.

## Declaration of Competing Interest

The authors have no financial conflicts of interest in regard to this research.

## Footnotes

Appendix A Supplementary data associated with this article can be found in the online version at doi:10.1016/j.dadr.2026.100449.
