# Supplementary material for: Associations between neighborhood disadvantage, cannabis use, and patient-reported outcomes among patients with cancer
Source: Drug Alcohol Depend Rep. 2026 May 23;19:100449. doi: 10.1016/j.dadr.2026.100449 (PMC13234724; doi:10.1016/j.dadr.2026.100449)
Supplement: Supplementary file 1 — Supplementary material [file mmc1.docx]

Supplemental Analyses

| **Supplemental Table 1.** | | | | | | |
| --- | --- | --- | --- | --- | --- | --- |
| Clinical Characteristics by Neighborhood Disadvantage | | | | | | |
| Characteristics | | Most Disadvantaged | Moderately Disadvantaged | Least Disadvantaged | Overall | *p*-value |
|  | | (n = 44) | (n = 34) | (n = 28) | (n = 106) |  |
| Stage n (%) | |  |  |  |  | .06 |
|  | 1 (Early) | 3 (6.8%) | 5 (14.7%) | 8 (28.6%) | 16 (15.1%) |  |
|  | 2 (Localized) | 8 (18.2%) | 10 (29.4%) | 5 (17.9%) | 23 (21.7%) |  |
|  | 3 (Regional Spread) | 12 (27.3%) | 9 (26.5%) | 3 (10.7%) | 24 (22.6%) |  |
|  | 4 (Distant Spread) | 13 (29.6%) | 8 (23.5%) | 11 (39.3%) | 32 (30.2%) |  |
|  | Other | 8 (18.2%) | 2 (5.9%) | 1 (3.6%) | 11 (10.4%) |  |
| Status n (%) | |  |  |  |  | .11 |
|  | In Remission | 9 (20.5%) | 11 (32.4%) | 5 (17.9%) | 25 (23.6%) |  |
|  | Current/Progressive | 11 (25.0%) | 5 (14.7%) | 11 (39.3%) | 27 (25.5%) |  |
|  | Stable/Responsive | 18 (40.9%) | 14 (41.2%) | 5 (17.9%) | 37 (34.9%) |  |
|  | Unknown | 6 (13.6%) | 4 (11.8%) | 7 (25.0%) | 17 (16.0%) |  |
| Treatment n (%) | |  |  |  |  | .51 |
|  | Surgery | 6 (13.6%) | 3 (8.8%) | 4 (14.3%) | 13 (12.3%) |  |
|  | Chemotherapy | 13 (29.6%) | 10 (29.4%) | 10 (35.7%) | 33 (31.1%) |  |
|  | Radiation | 13 (29.6%) | 10 (29.4%) | 11 (39.3%) | 34 (32.1%) |  |
|  | Immunotherapy | 6 (13.6%) | 2 (5.9%) | 2 (7.1%) | 10 (9.4%) |  |
|  | Hormone/Biological Therapy | 6 (13.6%) | 8 (23.5%) | 1 (3.6%) | 15 (14.2%) |  |
|  | Other | 0 (0.0%) | 1 (2.9%) | 0 (0.0%) | 1 (0.9%) |  |
| Cancer Type n (%) | |  |  |  |  | .21 |
|  | Breast | 11 (25.0%) | 13 (38.2%) | 7 (25.0%) | 31 (29.3%) |  |
|  | Gastrointestinal | 9 (20.5%) | 6 (17.7%) | 8 (28.6%) | 23 (21.7%) |  |
|  | Genitourinary/Gynecological | 5 (11.4%) | 8 (23.5%) | 3 (10.7%) | 16 (15.1%) |  |
|  | Head & Neck | 2 (4.6%) | 1 (2.9%) | 4 (14.3%) | 7 (6.6%) |  |
|  | Hematologic/Musculoskeletal | 6 (13.6%) | 3 (8.8%) | 0 (0.0%) | 9 (8.5%) |  |
|  | Neurological/Neuroendocrine | 2 (4.6%) | 0 (0.0%) | 0 (0.0%) | 2 (1.9%) |  |
|  | Thoracic | 5 (11.4%) | 3 (8.8%) | 4 (14.3%) | 12 (11.3%) |  |
|  | Other | 4 (9.1%) | 0 (0.0%) | 2 (7.1%) | 6 (5.7%) |  |

| **Supplemental Table 2.** | | | | | | | |
| --- | --- | --- | --- | --- | --- | --- | --- |
| Sociodemographic and Cannabis Characteristics by Income | | | | | | | |
| Characteristics | | | Low Income (< $35,000) | Lower-middle Income ($35,000 - $99,000) | Middle-upper Income (> $100,000) | Overall | *p*-value |
|  | | | (n = 40) | (n = 43) | (n = 22) | (n = 105) |  |
| Site n (%) | | |  |  |  |  | .17 |
|  | University at Buffalo (UB) | | 13 (32.5%) | 19 (44.2%) | 5 (22.7%) | 37 (35.2%) |  |
|  | University of Pennsylvania (UP) | | 9 (22.5%) | 12 (27.9%) | 10 (45.5%) | 31 (29.5%) |  |
|  | Thomas Jefferson University (TJU) | | 18 (45.0%) | 12 (27.9%) | 7 (31.8%) | 37 (35.2%) |  |
| Age mean (SD) | | | 54 (13) | 56 (11) | 53 (10) | 55 (12) | .45 |
| Sex n (female %) | | | 25 (62.5%) | 25 (58.1%) | 10 (45.5%) | 60 (57.1%) | .43 |
| Race n (black %) | | | 24 (60.0%) | 12 (27.9%) | 1 (4.6%) | 37 (35.2%) | **<.001** |
| State n (%) | | |  |  |  |  | .44 |
|  | Pennsylvania | | 25 (62.5%) | 19 (44.2%) | 14 (63.6%) | 58 (55.2%) |  |
|  | New York | | 13 (32.5%) | 19 (44.2%) | 5 (22.7%) | 37 (35.2%) |  |
|  | New Jersey | | 2 (5.0%) | 4 (9.3%) | 2 (9.1%) | 8 (7.6%) |  |
|  | Other | | 0 (0.0%) | 1 (2.3%) | 1 (4.6%) | 2 (1.9%) |  |
| Marital Status n (%) | | |  |  |  |  | **<.001** |
|  | Married or Cohabitating | | 9 (22.5%) | 25 (58.1%) | 19 (86.4%) | 53 (50.5%) |  |
|  | Divorced, Separated, or Widowed | | 15 (37.5%) | 11 (25.6%) | 2 (9.1%) | 28 (26.7%) |  |
|  | Never Married | | 16 (40.0%) | 7 (16.3%) | 1 (4.6%) | 24 (22.9%) |  |
| Education n (%) | | |  |  |  |  | **<.01** |
|  | High School Degree or Lower | | 11 (27.5%) | 6 (14.0%) | 4 (18.2%) | 21 (20.0%) |  |
|  | Some College/Technical School | | 23 (57.5%) | 17 (39.5%) | 6 (27.3%) | 46 (43.8%) |  |
|  | College Graduate or Greater | | 6 (15.0%) | 20 (46.5%) | 12 (54.6%) | 38 (36.2%) |  |
| Employment n (%) | | |  |  |  |  | **<.01** |
|  | Employed | | 3 (7.5%) | 10 (23.3%) | 12 (54.6%) | 25 (23.8%) |  |
|  | Retired or Disabled | | 31 (77.5%) | 25 (58.1%) | 6 (27.3%) | 62 (59.1%) |  |
|  | Unemployed |  | 5 (12.5%) | 7 (16.3%) | 2 (9.1%) | 14 (13.3%) |  |
|  | Other |  | 1 (2.5%) | 1 (2.3%) | 2 (9.1%) | 4 (3.8%) |  |
| Health Insurance n (%) | | |  |  |  |  | **<.001** |
|  | Employer Plan | | 2 (5.0%) | 18 (41.9%) | 16 (72.7%) | 36 (34.3%) |  |
|  | Private | | 1 (2.5%) | 4 (9.3%) | 2 (9.1%) | 7 (6.7%) |  |
|  | Medicaid/Medicare |  | 35 (87.5%) | 19 (44.2%) | 3 (13.6%) | 57 (54.3%) |  |
|  | Other |  | 2 (5.0%) | 2 (4.7%) | 1 (4.6%) | 5 (4.8%) |  |
| Income Satisfaction | | |  |  |  |  | **<.001** |
|  | Living Comfortably on Current Income | | 4 (10.0%) | 11 (25.6%) | 14 (63.6%) | 29 (27.6%) |  |
|  | Getting by on Current Income | | 15 (37.5%) | 21 (48.8%) | 3 (13.6%) | 39 (37.1%) |  |
|  | Finding it Difficult on Current Income | | 11 (27.5%) | 8 (18.6%) | 5 (22.7%) | 24 (22.9%) |  |
|  | Finding it Very Difficult on Current Income |  | 10 (25.0%) | 3 (7.0%) | 0 (0.0%) | 13 (12.4%) |  |
| Cannabis Use Frequency n (%) | | |  |  |  |  | .37 |
|  | A Few Times a Month |  | 5 (12.5%) | 1 (2.4%) | 2 (9.1%) | 8 (7.7%) |  |
|  | Once to Several Days a Week | | 9 (22.5%) | 14 (33.3%) | 8 (36.4%) | 31 (29.8%) |  |
|  | Daily or More | | 26 (65.0%) | 27 (64.3%) | 12 (54.6%) | 65 (62.5%) |  |
| Used Multiple Cannabis Routes n (yes %) | | | 11 (27.5%) | 11 (25.6%) | 9 (40.9%) | 31 (29.5%) | .41 |
| Went to Multiple Cannabis Sources n (yes %) | | | 6 (15.0%) | 9 (20.9%) | 2 (9.1%) | 17 (16.2%) | .46 |
| Cannabis Dependence mean (SD) | | | 7 (4.0) | 6 (3.0) | 6 (2.0) | 6 (3.0) | .66 |
| Opioid Prescription Receipt n (yes %) | | | 30 (75.0%) | 29 (67.4%) | 13 (59.1%) | 72 (68.6%) | .43 |
| **Note.** Bolded results represent associations significant at the *p* < .05 level. No reported income or education data for one participant. | | | | | | | |

| **Supplemental Table 3.** | | | | | | |
| --- | --- | --- | --- | --- | --- | --- |
| Clinical Characteristics by Income | | | | | | |
| Characteristics | | Low Income (< $35,000) | Lower-middle Income ($35,000 - $99,000) | Middle-upper Income (> $100,000) | Overall | *p*-value |
|  | | (n = 40) | (n = 43) | (n = 22) | (n = 105) |  |
| Stage n (%) | |  |  |  |  | .13 |
|  | 1 (Early) | 5 (12.5%) | 5 (11.6%) | 6 (27.3%) | 16 (15.2%) |  |
|  | 2 (Localized) | 14 (35.0%) | 8 (18.6%) | 1 (4.6%) | 23 (21.9%) |  |
|  | 3 (Regional Spread) | 9 (22.5%) | 10 (23.3%) | 5 (22.7%) | 24 (22.9%) |  |
|  | 4 (Distant Spread) | 7 (17.5%) | 16 (37.2%) | 8 (36.4%) | 31 (29.5%) |  |
|  | Other | 5 (12.5%) | 4 (9.3%) | 2 (9.1%) | 11 (10.5%) |  |
| Status n (%) | |  |  |  |  | .07 |
|  | In Remission | 13 (32.5%) | 10 (23.3%) | 2 (9.1%) | 25 (23.8%) |  |
|  | Current/Progressive | 10 (25.0%) | 8 (18.6%) | 9 (40.9%) | 27 (25.7%) |  |
|  | Stable/Responsive | 14 (35.0%) | 14 (32.6%) | 9 (40.9%) | 37 (35.2%) |  |
|  | Unknown | 3 (7.5%) | 11 (25.6%) | 2 (9.1%) | 16 (15.2%) |  |
| Treatment n (%) | |  |  |  |  | .18 |
|  | Surgery | 7 (17.5%) | 5 (11.6%) | 1 (4.6%) | 13 (12.4%) |  |
|  | Chemotherapy | 11 (27.5%) | 10 (23.3%) | 12 (54.6%) | 33 (31.4%) |  |
|  | Radiation | 14 (35.0%) | 13 (30.2%) | 7 (31.8%) | 34 (32.4%) |  |
|  | Immunotherapy | 2 (5.0%) | 5 (11.6%) | 2 (9.1%) | 9 (8.6%) |  |
|  | Hormone/Biological Therapy | 6 (15.0%) | 9 (20.9%) | 0 (0.0%) | 15 (14.3%) |  |
|  | Other | 0 (0.0%) | 1 (2.3%) | 0 (0.0%) | 1 (1.0%) |  |
| Cancer Type n (%) | |  |  |  |  | .64 |
|  | Breast | 12 (30.0%) | 14 (32.6%) | 5 (22.7%) | 31 (29.5%) |  |
|  | Gastrointestinal | 9 (22.5%) | 8 (18.6%) | 6 (27.3%) | 23 (21.9%) |  |
|  | Genitourinary/Gynecological | 6 (15.0%) | 5 (11.6%) | 4 (18.2%) | 15 (14.3%) |  |
|  | Head & Neck | 1 (2.5%) | 2 (4.7%) | 4 (18.2%) | 7 (6.7%) |  |
|  | Hematologic/Musculoskeletal | 5 (12.5%) | 3 (7.0%) | 1 (4.6%) | 9 (8.6%) |  |
|  | Neurological/Neuroendocrine | 1 (2.5%) | 1 (2.3%) | 0 (0.0%) | 2 (1.9%) |  |
|  | Thoracic | 4 (10.0%) | 6 (14.0%) | 2 (9.1%) | 12 (11.4%) |  |
|  | Other | 2 (5.0%) | 4 (9.3%) | 0 (0.0%) | 6 (5.7%) |  |
